# The complex nature of lncRNA-mediated chromatin dynamics in multiple myeloma

**DOI:** 10.3389/fonc.2023.1303677

**Published:** 2023-12-11

**Authors:** Patrick Nylund, Berta Garrido-Zabala, Antonia Kalushkova, Helena Jernberg Wiklund

**Affiliations:** Science for Life Laboratory, Department of Immunology, Genetic and Pathology, Rudbeck Laboratory, Uppsala University, Uppsala, Sweden

**Keywords:** lncRNA, chromatin regulation, multiple myeloma, epigenetics, RNA modifications, haematological malignancies

## Abstract

Extensive genome-wide sequencing efforts have unveiled the intricate regulatory potential of long non-protein coding RNAs (lncRNAs) within the domain of haematological malignancies. Notably, lncRNAs have been found to directly modulate chromatin architecture, thereby impacting gene expression and disease progression by interacting with DNA, RNA, and proteins in a tissue- or condition-specific manner. Furthermore, recent studies have highlighted the intricate epigenetic control of lncRNAs in cancer. Consequently, this provides a rationale to explore the possibility of therapeutically targeting lncRNAs themselves or the epigenetic mechanisms that govern their activity. Within the scope of this review, we will assess the current state of knowledge regarding the epigenetic regulation of lncRNAs and how, in turn, lncRNAs contribute to chromatin remodelling in the context of multiple myeloma.

## Introduction

Multiple myeloma (MM) is a heterogeneous haematological malignancy characterized by the clonal expansion of malignant plasma cells within the bone marrow (1). It represents the second most prevalent haematological malignancy and it is marked by complex genetic aberrations, including chromosomal translocations, copy number alterations, and somatic mutations, affecting pathways critical to cell cycle regulation, DNA repair, and epigenetic modulation (2–4). Treatment strategies include high-dose chemotherapy regimens, autologous stem cell transplantation, as well as targeted therapies such as proteasome inhibitors and immunomodulatory agents. Despite these therapeutic innovations, disease relapse and drug resistance remain as substantial challenges (5). Thus, treatment of MM is clinically challenging and new therapeutic interventions are required. Prior studies, by us and others, have suggested that the epigenetic machinery plays a crucial role in MM pathogenesis, including aberrant DNA methylation and abnormal histone modification patterns (6–14). Furthermore, more recently, dysregulation of long non-protein coding RNAs (lncRNAs) has been suggested to contribute to MM pathogenesis, patient outcome and drug resistance (15–17). Additionally, dysregulation of lncRNAs has been shown to contribute to disease progression by influencing critical pathways involved in proliferation, apoptosis, immune response, and drug resistance (18, 19). Unravelling the complex network of lncRNA-mediated molecular mechanisms could therefore unveil novel therapeutic targets and diagnostic markers in MM.

lncRNAs represent the largest group of non-protein coding RNAs, however, to date their functions remain largely unexplored. lncRNAs are transcripts exceeding 200 nucleotides in length, and their transcriptional regulation mirrors that of protein-coding genes, including processes such as histone modifications, chromatin compaction, and chromatin remodelling. The biogenesis of lncRNAs encompasses a spectrum of events, including 5’ capping, splicing, variation in exon and intron dimensions, and the addition of polyadenylation (poly(A)+) tails. Notably, features like poly(A)+ tails and 5’ capping play fundamental roles in determining the transcript stability of lncRNAs. In contrast to messenger RNAs (mRNAs), lncRNAs transcripts exhibit a diminished steady-state, as they are commonly less evolutionary conserved (20). lncRNAs can be transcribed from multiple genomic locations, including promoters, enhancers, intergenic regions, as well as in bidirectional and antisense directions. Typically residing within the nucleus, lncRNAs tend to manifest pronounced cell and tissue specificity (21). In addition, a substantial fraction i.e., 81% of lncRNAs, exhibit a limited degree of evolutionary conservation, while 3% of lncRNAs manifest ultra-conservation (22).

**Figure 1:**
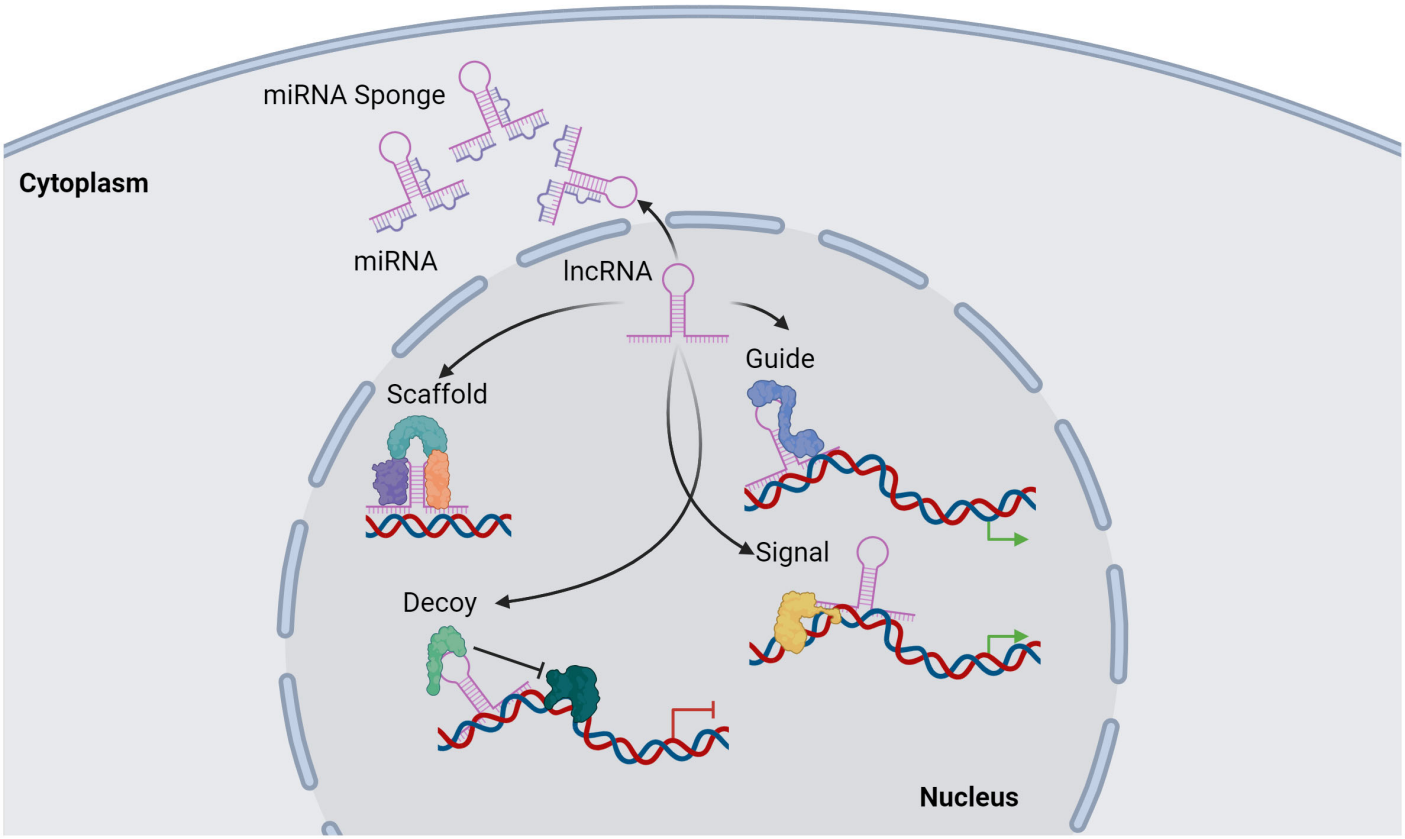
Overview of lncRNA functions. lncRNAs can regulate transcription by acting as a scaffold by binding proteins together in a complex structure. A secondary function of a lncRNA is as a guide of proteins or other molecules to target genomic location. lncRNAs can directly bind to genomic regions within the genome to transduce signal activation of DNA-bound molecules. Furthermore, a lncRNA can act as a decoy, preventing different molecules such as proteins to bind to targeted genomic regions. In addition, lncRNAs can regulate miRNAs function by acting as a miRNA sponge, preventing miRNA-mRNA binding, thus inhibiting mRNAs degradation. Image was created with biorender.com.

**Figure 2:**
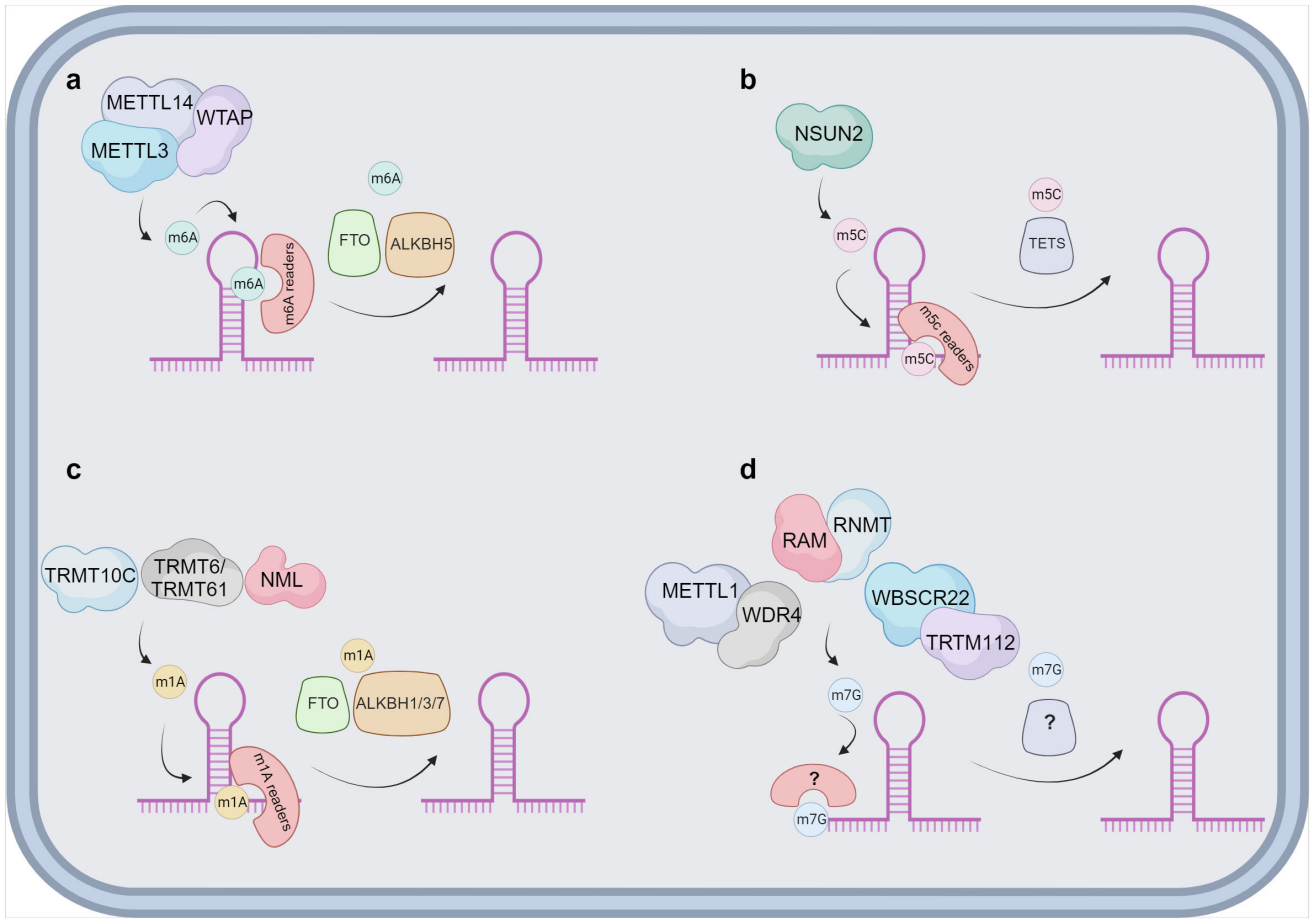
Proposed overview of RNA modifications on lncRNA. Schematic overviews are provided for the different lncRNAs modifications. **(A)** The m6A modification is deposited by a protein complex constituted mainly by METTL3, METTL14 and the cofactor WTAP. There are multiple m6A readers identified, namely YTHDF1/2/3, IGF2BP1/2/3, HNRNPs and ZC3H13. The demethylation of m6A is catalysed by FTO and ALKBH5. **(B)** NSUN2 has been reported as the sole writer of the m5C modification in lncRNAs. The m5C can be identified by ALYREF and YBX1 and it is speculated that the removal is catalysed by TETs. **(C)** m1A is suggested to be deposited by TRMT10C, TRMT6/61 and NML on lncRNAs. Multiple readers have been identified in other types of RNAs, as YTDHF1-3 and YTHDC1 and the removal is mainly associated to FTO and ALKBH1/3/7. **(D)** The exact manner of how m7G modification is deposited on lncRNAs is currently unknown. Three different complexes can take the role of a writer, METTL1 and WDR4, RAM and RNMT or WBSCR22 and TRTM112. Currently both readers and erasers on m7G are unknown and represented with ? in the figure. Image was created with biorender.com.

Functionally, lncRNAs perform a diverse array of functions both within the nucleus and the cytoplasm. These molecules regulate gene expression by engaging in intricate interactions with RNA, DNA and proteins, including chromatin-modifying enzymes. Within the nuclear domain, lncRNAs have been categorized into four fundamental archetypes: signal, decoy, guide, and scaffold lncRNAs (**Figure 1**). Signal lncRNAs respond to specific stimuli, promoting integration of signals for the transcription of targeted genes (23). Decoy lncRNAs, on the other hand, can bind proteins, such as transcription factors and chromatin modifiers, resulting in transcriptional control by impeding the binding capacity to their targets (24). Guide lncRNAs have the ability to reposition ribonucleoprotein complexes to designated loci, both in *cis* and in *trans*, thereby altering the gene expression patterns. Finally, scaffold-associated lncRNAs engage in temporally and spatially regulated interactions with DNA, different types of RNAs and proteins, thereby bolstering the stability of complexes involved in either transcriptional activation or suppression. Additionally, lncRNAs operate as microRNA (miRNA) sponges, sequestering miRNAs to avert mRNA degradation (25). To date, various lncRNAs have been described to localize with chromatin, where they interact with different chromatin-associated proteins to promote or repress their binding potential to specific DNA locations. These chromatin-associated lncRNAs have been implicated in MM pathogenesis and disease outcome. In addition, lncRNAs do not only act as regulators of the epigenetic landscape but can also themselves be epigenetically regulated by DNA and chromatin modifications as well as by RNA modifications, referred to as the epitranscriptomics. Among these, RNA modifications such as, N6-methyladenosine (m6A), N1-methyladenosine (m1A), 5-methylcytosine (m5C), and 7-methylguanosine (m7G), play a crucial role in regulating various aspects of lncRNA function, structure, stability, localization, and lncRNA-mediated interactions (26–34) (**Figure 2**).

We and others have shown the importance of lncRNAs in chromatin remodelling and the impact they have on MM patient outcome (6, 15–17). In fact, aberrant lncRNA expression has been demonstrated to have an oncogenic role in MM pathogenesis and progression (35, 36). In this review, we present an overview of the role of lncRNAs in the context of MM through their epigenetic regulation and functional effects on chromatin remodelling.

## The functional impact of lncRNAs in epigenetic regulation

lncRNAs have been suggested to affect multiple layers of cellular function, encompassing processes such as cellular biogenesis of macromolecules, differentiation, gene expression and chromatin remodelling. The recent establishment of a comprehensive genome-wide lncRNA-chromatin interactome has provided insight into the intricate orchestration of chromatin compaction by lncRNAs, subsequently impacting gene expression patterns (37–39). Notably, the functional implications of lncRNA-mediated chromatin remodelling in the context of cancer have gained considerable interest, however a summary within the domain of MM is currently lacking.

### Epigenetic regulation of lncRNAs by DNA methylation and histone modifications

The expression of lncRNAs can be regulated by different epigenetic machineries, such as DNA methylation (40). DNA methylation plays an important role in regulating cell-type specific gene expression. The DNA methylation process consists of the deposition of methyl groups to the 5-carbon position of cytosine in a CpG dinucleotide, resulting in gene suppression when located along the promoter or transcription start site and gene transcription when found in the gene body. This process is catalysed by the DNA methyltransferases, DNMT1 and DNMT3A/B and can be reversed by the DNA demethylase enzymes TET1-3 (41). Disrupted DNA methylation has been shown to promote carcinogenesis and disease progression in multiple cancers (3, 42, 43). In fact, it has previously been suggested that promoter DNA hypermethylation is accountable for decreased expression of 35 lncRNAs in hepatocellular carcinoma (40, 44). Furthermore, patients with lower expression of these lncRNAs had increased expression of the DNA methyltransferase genes *DNMT1*, *DNMT3A*, and *DNMT3B*. In contrast, patients with higher expression of this panel of lncRNAs, exhibited lower expression of the DNA methyltransferases (40). Li et al. reported that the lncRNA *BM742401*, defined as a tumour suppressor in gastric cancer and chronic lymphocytic leukaemia, undergoes silencing in MM cell lines due to promoter hypermethylation. Notably, decreased *BM742401* levels enhanced MM cell migration, while in newly diagnosed MM patients, silencing by elevated DNA methylation levels in the promoter of the lncRNA *BM742401* correlated with poor overall survival. This underscores the significant impact that epigenetic regulation of lncRNAs can exert on disease progression (45). Similarly, DNA methylation-mediated silencing of the lncRNA *KIAA0495* has been reported in MM cell lines, although it was not found to be relevant for the progression of the disease (46) (**Table 1**).

**Table 1:** Function and clinical implications of lncRNAs in multiple myeloma.

lncRNA	Expression	Function	Downstream effects	Prognosis	Reference
*BM742401*	Downregulated	Unknown	Promote cell migration	Poor OS	(45)
*KIAA0495*	Downregulated	Unknown	Unknown	Not involved	(46)
*PVT1*	Upregulated	PRC2 recruiter	Silencing of tumor suppressor genes & pro-apoptotic genes	Poor OS	(6)
*ANRIL*	Upregulated	Guide for PRC1/2	Resistance to bortezomib	Poor OS	(47)
*H19*	Upregulated	miRNA sponge & activator of BRD4	Imbalance of osteogenesis/osteolysis	Poor OS	(48)
*CRNDE*	Upregulated	Unknown	Proliferation through IL6 signalling	Poor OS	(49)
*MIAT*	Upregulated	Unknown	Resistance to bortezomib	Poor OS	(50)
*HOTAIR*	Upregulated	Activation of NF-κB & JAK2/STAT3 signalling	Resistance to dexamethasone	Unknown	(51)
*RROL*	Unknown	Chromatin scaffold	Promote cell growth	Unknown	(52)
*AIR*	Upregulated	Unknown	Unknown	Unknown	(53)
*HOXB-AS1*	Upregulated	mRNA stabilizer	Unknown	Unknown	(54)
*DARS-AS1*	Unknown	Unknown	Promoting the mTOR pathway	Unknown	(55)
*MALAT1*	Upregulated	Scaffold for protein complexes & miRNA sponge	Increased proliferation & reduction of pro-apoptotic gene expression	Poor OS	(25, 56, 57)
*NEAT1*	Upregulated	Unknown	Chemotherapeutic resistance	Poor OS	(25, 58)
*GAS5*	Upregulated	Unknown	Unknown	Poor OS	(25)

An additional level of transcriptional regulation is through chromatin compaction. DNA is packed into chromatin fibres wrapped around a histone octamer, ultimately forming a nucleosome. The nucleosome consists of the four histone proteins H2A, H2B, H3 and H4. Each histone protein has in its N-terminal domain a histone tail that can be reversibly subjected to methylation, acetylation, phosphorylation, ubiquitination, sumoylation and histone tail clipping which control chromatin compaction, thus either promoting or inhibiting transcription factor binding, DNA repair, replication and genomic recombination. The majority of studies have concentrated on examining histone modifications related to protein coding genes and non-protein coding genes such as miRNAs (59–62). Consequently, additional research is warranted to elucidate the influence of histone modifications on lncRNAs’ regulation and the potential implications in various diseases, including MM.

### Interplay between lncRNAs and chromatin modifiers

Although, data is largely lacking how regulation of lncRNAs by the deposition of histone modifications may directly influence their expression, there is now emerging data indicating that lncRNAs may act as recruiters, guides and scaffolds for protein complexes including chromatin modifiers, thus epigenetically influencing the expression of other genes. Prior studies have shown that PRC2-mediated gene silencing is important for MM pathogenesis and disease progression, both *in vivo* and *in vitro* (7, 8, 10, 43). Furthermore, several lncRNAs have been suggested to regulate the enzymatic activity of PRC2 by binding to the catalytic subunit EZH2. Moreover, lncRNAs can modulate PRC2 activity by acting as a complex recruiter to target genomic locations. For instance, the lncRNA *PVT1* was recently described to be overexpressed in primary MM patient samples and associated with poor prognosis, a seemingly independent feature from patients’ cytogenetic background (6). Moreover, *PVT1* was shown to interact directly with EZH2, facilitating recruitment of PRC2 to target genomic loci and transcriptional repression of genes associated with pro-apoptotic and tumour suppressor functions (6) (**Table 1**). Similarly to the function of *PVT1*, the lncRNA *ANRIL*, was described to exert a guiding function for PRC1 and PRC2 DNA binding in MM and was demonstrated to promote resistance to conventional therapies such as bortezomib by guiding PRC2 to promote gene silencing of the tumour suppressor gene *PTEN.* High expression of *ANRIL* has been associated with poor overall survival in MM (47) (**Table 1**). Furthermore, upregulation of the lncRNA *H19* correlates with worse prognosis and promotes the imbalance of osteogenesis and osteolysis in MM by acting as a miRNA sponge, resulting in upregulation of E2F7, which is a transcriptional activator of EZH2 and thus affecting the suppression of *PTEN* (48). In addition, increased *H19* activity has been shown to activate the chromatin reader protein BRD4 in MM (63). BRD4 is a well-known epigenetic reader of acetylated lysine and assists in the transmission of epigenetic memory during cell division (64, 65). BRD4 has been identified as a therapeutic vulnerability and potential target in MM (66) (**Table 1**).

The lncRNA *CRNDE* epigenetically regulates the transcription of *DUSP5* and *CDKN1A* in solid tumours by facilitating PRC2 recruitment (67). Overexpression of *CRNDE* has been described to be associated with poor prognosis by regulating proliferative capacity through IL6 signalling in MM, however, no direct interaction between *CRNDE* and PRC2 has been proven (49). Recruitment of the histone H3 lysine 4 methyltransferase MLL has been suggested to occur through the binding to the lncRNA *MIAT*, which can then guide MLL to the promoter region of the collagen degradation enzyme *MMP9*. Inhibition of *MIAT* resulted in the loss of transcriptional activity of *MMP9*, which is suggested to reduce proliferative capacity and cell migration in non-small cell lung cancer (68). In MM*, MIAT is* overexpressed and has been associated with sensitivity to bortezomib treatment (50) (**Table 1**).

Interestingly, additional lncRNAs have been suggested to play important roles in chromatin regulation. The lncRNA *HOTAIR* has been demonstrated to bind to the PRC2 complex and can further interact with the TF-silencing complex formed by LSD1/CoREST/repressor element 1, promoting gene repression (69). In addition, *HOTAIR* may function as a stabilizing component of PRC2, as well as a scaffold for complex-complex interactions (69). In MM, *HOTAIR* has been described to be upregulated in primary patient samples and to contribute to the oncogenic activation of the JAK2/STAT3 signalling pathway (51) (**Table 1**). Similarly, the MIR17HG-derived lncRNA, *RROL*, has been demonstrated to act as a chromatin scaffold for protein interactions and to promote MM cell growth (52). lncRNAs such as *AIR* and *HOXB-AS1* have been described to have a guiding function through which they recruit the histone methyltransferases G9a and SET1/MLL to target locations to induce gene repression or activation, respectively (53, 70). Interestingly, *HOXB-AS1* has been described to be upregulated in MM, acting as a stabilizer for mRNA (54) (**Table 1**). In another aspect of epigenetic regulation, *DARS-AS1* promotes the recruitment of the histone methyltransferases METTL3 and METTL14 to *DARS* mRNA to induce m6A modification and enhance translation in cervical cancer (71). In MM, *DARS-AS1* has been described to regulate HIF-1α in promoting the mTOR pathway (55) (**Table 1**).

Increased expression of the lncRNAs *GAS5*, *MALAT1* and *NEAT1* in MM patients, is associated with poor outcome and worse overall survival (25) (**Table 1**). *GAS5* has the ability to act as decoy for different molecules by functioning as a DNA mimic, thus preventing DNA motif binding (72). One of the most abundant and most studied lncRNAs is *MALAT1* which has been implicated in various functions during MM pathogenesis by acting as a scaffold for proteins involved in DNA repair (56) and as a miRNA sponge (57). Interestingly it has also been described to promote gene silencing by PRC2 recruitment in various cancers (73–75). Recent studies in colorectal cancer have suggested that the lncRNA *NEAT1* promotes histone H3 lysine 27 acetylation in genes associated with stemness (76). In addition, *NEAT1* has further been implicated in lung cancer by recruiting DNMT1 to the promoter regions of genes regulating cytotoxic T-cell infiltration. In fact, inhibition of *NEAT1* leads to loss of DNMT1 binding to these promoter regions and thus activating gene expression (77). In MM, overexpression of *NEAT1* has been associated with poor patient outcome. In addition, and further supporting a clinical relevance, inhibition of *NEAT1* promoted increased sensitivity to chemotherapeutic treatment (58) (**Table 1**).

### Epigenetic regulation of lncRNAs by RNA modifications

RNA modifications on lncRNAs may influence their stability, subcellular localization, and interactions with DNA, proteins and other RNA molecules. These modifications can also affect lncRNA regulation and contribute to their reported functional diversity (33). Dysregulation of RNA modifications on lncRNAs has been associated with various diseases, including MM (33, 78–80).

The deposition of the N6-methyladenosine (m6A) mark may give rise to structural changes in lncRNAs, thus modifying lncRNA-protein interactions. Additionally, the m6A modification can modulate gene transcription, influence the subcellular localization of lncRNAs and regulate lncRNAs’ stability (81–84). There is an interdependent connection between the m6A modification and lncRNAs. Notably, lncRNAs have the ability to influence the stability and degradation of enzymes involved in m6A, as well as facilitate their integration into protein complexes (85–87). One example of this function is the lncRNA *FEZF1-AS1*, the knockdown of which led to an increased apoptosis by regulating the signalling of IGF2BP1, an m6A reader protein, in MM (88). Furthermore, dysregulation of m6A-related enzymes has been associated with disease progression, enhancing tumour growth and cell proliferation in MM (89–95). Significantly, m6A studies in MM showed a correlation between exosome-induced drug resistance and high levels of m6A on the lncRNAs *LOC606724* and *SNHG*. Wang et al. identified METTL7A as an additional component of the m6A methyltransferase complex and described how its regulation is mediated by EZH2. Depletion of EZH2 simultaneously reduced METLL7A protein methylation levels, thus altering the m6A levels on the lncRNAs *LOC606724* and *SNHG* (96). Studies in prostate cancer show that high levels of m6A on *NEAT1* have been associated with bone metastasis (79, 97). Although no studies of m6A on *NEAT1* have been performed in MM, high expression of *NEAT1* in patients have been correlated with poor prognosis (98). In addition, *NEAT1* can enhance the preservation of DNA integrity, thus promoting survival of MM cells (99). Moreover, knockdown of *NEAT1* improved dexamethasone drug response in MM cell lines (100).

5-methylcytosine (m5C) has previously been described to exert important functions on DNA and has also been found to occur on RNA (78). The biological impact of RNA m5C primarily affects RNA localization, stability and transcription efficiency (101). Interestingly, NSUN2 has been reported as the sole writer of the m5C mark on lncRNAs (79). In MM, dysregulated deposition of RNA m5C has been correlated with disease progression and immune microenvironment regulation (102). Furthermore, recent studies have elucidated the importance of this modification in various other cancer types, including lung adenocarcinoma, pancreatic cancer, and colon cancer (79, 103–105).

Modifications of lncRNAs also include the deposition of N1-methyladenosine (m1A), which alters RNA secondary and tertiary structure, subsequently affecting its capacity to interact with RNA binding proteins. However, the function of m1A in lncRNAs is not fully elucidated, and the m1A modification has so far only been reported in the lncRNA *MALAT1* (80, 106). Despite the absence of studies focusing on the m1A modification in MM, as previously mentioned, *MALAT1* overexpression is correlated with worse prognosis, and the oncogenic role of *MALAT1* in promoting MM tumorigenesis has been widely studied (35, 56, 107). *MALAT1* dysregulation in MM has been associated with a wide range of processes including cell proliferation, DNA repair mechanisms, metastasis, drug resistance, and angiogenesis pathways (57, 107–109). Nonetheless, if these functions are mediated by chromatin remodelling and regulated via RNA modifications remains to be further investigated.

The N7-methylguanosine (m7G) modification is predominantly found at the 5´cap of mRNA, ribosomal RNAs (rRNAs) and transfer RNAs (tRNAs). However, the impact of m7G on lncRNAs remains uncertain, probably attributed to the absence of 5´cap on less conserved lncRNAs (110, 111). Nevertheless, Yang et al. constructed the first model based on eight m7G-related lncRNAs to predict patient prognosis in colon cancer (112). Similarly, RNA m7G MeRIP-seq uncovered the significance of m7G-enriched lncRNAs in acute myeloid leukemia cells and unravelled a potential role of this modification in modulating gene expression, thereby enhancing drug resistance (111). However, the role of m7G modification in MM remains at present unknown.

## Discussion

The pathogenic impact of lncRNAs in MM and other haematological malignancies is unravelling. Recently, there have been large sequencing efforts in various cancers including MM that have suggested a clinical importance of lncRNAs. In MM, lncRNAs have been implicated in clinically relevant elements such as disease development, progression, drug resistance and patient outcome (25). Studies on the epitranscriptomics of lncRNAs through the addition of methyl groups to the lncRNA transcripts have gained increased attention and have furthered added an additional level of complexity to how lncRNAs contribute to cellular processes, such as RNA stability, translational efficiency of mRNAs and protein complex formation. However, the exact nature of these modifications needs to be further investigated in the context of MM. Moreover, not only can the expression of lncRNAs be epigenetically regulated but can in turn regulate chromatin modifying enzymes. Although lncRNA-chromatin interactions are clearly more dynamically investigated in some areas, such as in the recently shown context of PRC2 recruitment, deep functional evaluation of lncRNAs in MM is still lacking. It is apparent that this field is underdeveloped and a complete picture of how lncRNAs impact the pathophysiological processes in MM remains uncertain. While their functions continue to unfold, targeting lncRNAs arises as compelling innovative treatment option in cancer, including MM.

## Author contributions

PN: Conceptualization, Project administration, Visualization, Writing – original draft, Writing – review & editing. BG-Z: Conceptualization, Project administration, Visualization, Writing – original draft, Writing – review & editing. AK: Supervision, Writing – review & editing. HW: Funding acquisition, Supervision, Writing – review & editing, Conceptualization, Visualization.
